# IMPLEMENTING A HYBRID TELEHEALTH HOME-BASED TRANSITIONAL CARE PROGRAM: PRE-IMPLEMENTATION CHALLENGES

**DOI:** 10.1093/geroni/igac059.2179

**Published:** 2022-12-20

**Authors:** Lauren Penney, Teresa Damush, Dawn Bravata, Cathy Schubert, Alaina Preddie, Sean Baird, Ashley Schwartzkopf

**Affiliations:** South Texas Veterans Health Care System, San Antonio, Texas, United States; Richard L. Roudebush VA Medical Center, Indianapolis, Indiana, United States; Richard L. Roudebush VA Medical Center, Indianapolis, Indiana, United States; Richard L. Roudebush VA Medical Center in Indianapolis, Indianapolis, Indiana, United States; Richard L. Roudebush VA Medical Center, Indianapolis, Indiana, United States; Richard L. Roudebush VA Medical Center, Indianapolis, Indiana, United States; Richard L Roudebush VA Medical Center, Indianapolis, Indiana, United States

## Abstract

The reach of home-based clinical programs for medically complex, older adults can be limited by geographic scope. Implementing telehealth versions can expand reach but create constraints for comprehensive assessments and technological barriers for users. We describe challenges and lessons learned during the pre-implementation period for the randomized trial of a hybrid video -modality of the Geriatrics Resources for Assessment and Care for Elders (GRACE) Program at the Indianapolis Veterans Health Care System. In TeleGRACE, a health technician makes home visits to facilitate clinical activities (e.g., medication reconciliation) using telehealth technology for a clinical team (social worker, nurse practitioner) who conduct the visits remotely. Data used in this one-year pre-implementation evaluation included: periodic reflections with the clinical and evaluation staff, planning and interdisciplinary team meeting fieldnotes, and interviews with clinical team members. Data were summarized by selected constructs from the Consolidated Framework for Implementation Research; implementation challenges and problem solving were identified. Pre-implementation challenges occurred in: assuring assessment devices worked correctly for and were trusted by staff (e.g., connectivity, virtual stethoscope), technician fit with existing GRACE team, ensuring technician welfare (e.g., COVID-19 exposure, guns in the home), caseload balance amid staffing shortages, travel logistics, and sampling to adequately power the trial. Building on an existing strong team dynamic and a culture of feedback for quality improvement, challenges were addressed through pilot-testing, monitoring for barriers and impacts, and group reflecting conversations. Adaptations to the initial plan resulted in a more focused and targeted implementation effort to test the model and its effectiveness.

